# Oxford classification of IgA nephropathy and C4d deposition; correlation and its implication

**Published:** 2015-12-02

**Authors:** Ashutosh Rath, Rohit Tewari, Satish Mendonca, Sonia Badwal, Vijay Shrawan Nijhawan

**Affiliations:** ^1^Department of Pathology, Armed Forces Medical College, Pune, Maharashtra, India; ^2^Department of Internal Medicine (Nephrology), Command Hospital, Pune, Maharashtra, India; ^3^Department of Pathology, Army Hospital (Research and Referral), New Delhi, India

**Keywords:** IgA nephropathy, Oxford classification system, Immunohistochemistry

## Abstract

**Introduction:** IgA nephropathy (IgAN) is well known to be the most common form of primary glomerulonephritis throughout the world. The histopathological changes are wide and varied as brought out by the various classification systems like the Haas and Oxford systems. C4d is a well-known biomarker of the complement cascade and has recently been implicated in certain native renal diseases. We attempted to characterize C4d deposition in IgAN and correlate this with histopathology by the Oxford classification system.

**Patients and Methods:** This retrospective study included renal biopsies of 15 cases of IgAN diagnosed on histopathology and immunofluorescence over a period of 2 years. Demographic parameters of age and sex were reviewed. The Oxford classification system was applied to score the cases and immunohistochemistry for C4d was done on all cases to characterize staining pattern and intensity and was correlated with Oxford classification.

**Results:** On histological examination, the cases showed various combinations of lesions ranging from M0E0S0T0 to M1E1S1T1. C4d deposition was found to be occurring mainly in mesangial location (12/15 cases, 80%). Forty percent cases showed C4d deposition in the glomerular capillary walls in a segmental fashion and 26.67% showed global pattern. Other patterns of deposition were arteriolar (53.33%), in peritubular capillaries (26.67%) and in tubular epithelium (20%).

**Conclusion:** On comparing the various patterns of deposition of C4d with the four variables of the Oxford classification system, we found that segmental and global deposition of C4d correlated best with endocapillary proliferation.

**Implication for health policy/practice/research/medical education:**

We correlated C4d deposition with Oxford classification of IgA nephropathy (IgAN) and found endocapillary proliferation (E) to be the best parameter. Due to lesser number of cases, further larger scale study is needed to substantiate this. This would help in directed therapy against complement system.

## Introduction

IgA nephropathy (IgAN) first became recognized as a distinct entity in 1968, when Berger and Hinglais reported a cohort of patients with persistent microscopic haematuria, episodes of macroscopic haematuria in some that were often associated with a sore throat, mild to moderate proteinuria without the nephrotic syndrome, and normal renal function in most. It is an immune complex mediated disease defined by the presence of either dominant or co-dominant deposits of IgA, predominantly in the glomerular mesangium. Mesangial IgA deposition incites an inflammatory process that leads to mesangial proliferation and interstitial damage that slowly progresses to sclerosis and end-stage renal disease (ESRD) in approximately 40% of cases (1,2).

The pathogenesis of IgAN has been revolving around the complement activation. Earlier studies have shown the alternative pathway of complement activation to be induced by IgA (3). Later studies indicated the presence of mannose binding lectin (MBL) in association with IgA in the mesangial area of patients with IgAN (4). This was hypothesized on the basis of in vitro studies which found the evidence for C4 activation in the absence of C1q deposition (5).

C4d is a well-known biomarker of the complement cascade. It is derived from cleavage of the labile thioester bond of C4b. This cleavage provides C4d a covalent bond which helps C4d to anchor to nearby cells where immune complexes are deposited. Antibodies dissociate naturally because of relatively weak hydrostatic and Van der Waals forces between antigens and antibodies, whereas covalent bond of C4d has a much longer half-life. For this reason, C4d serves as a footprint for complement activation (6). The utility of C4d in the identification of antibody-mediated rejection (AMR) has been known since its incorporation in the Banff classification in 2003 (7). Recently, many researchers have turned their attention to C4d deposition in native renal diseases. Xing et al investigated that complement activation is involved in renal damage of pauci-immune crescentic glomerulonephritis (8). Espinosa-Hernandez et al suggested that C4d is a useful tool for the differential diagnosis of membranous nephropathy and minimal change disease (9).

Several classification systems have been proposed for stratifying the risk of progression in IgAN. To produce evidence based international consensus classification of IgAN, Renal Pathology Society and the International IgA Nephropathy Network set up an international group of pathologists which came up with Oxford classification. The Oxford study found that there were four lesions that were independently predictive of clinical outcome (10). This classification system was found to be better than the earlier proposed classifications. However, the Oxford study is unable to provide information on the question that which features are able to predict response to therapy.

## Objectives

In this study we attempted to correlate the Oxford classification and C4d deposition in IgAN cases and tried to arrive at its clinical implication.

## Patients and Methods

### Subjects

All the kidney biopsies reported as IgAN between 1 January 2010 to 31 December 2013 were included in the study. Clearance was taken from Institutional ethical committee. Fifteen renal biopsies diagnosed as IgAN were included in the study.

### Histopathological study

In all cases the biopsy was fixed in formalin and routinely processed. Three micron serial sections of these biopsies were studied with Hematoxylin & Eosin, PAS, PASM and Masson Trichrome. Tissue for immunofluorescence for these biopsies was transported in saline medium and frozen sections were stained with FITC conjugated antibodies to IgG, IgA, IgM, C3, and C1q to demonstrate the dominant or co-dominant deposition of IgA in glomerular mesangium.

### Oxford classification system

On light microscopy, biopsies were evaluated by the Oxford system of classification of IgAN and given a MEST score. Mesangial proliferation (M) was defined as presence of more than 4 nuclei in a single mesangial segment. Endocapillary hypercellularity (E) was considered to be present when there was increased number of cells within glomerular capillary lumina, causing narrowing of the lumina. Sclerosis was defined as obliteration of the capillary lumen by increased extracellular matrix, with or without hyalinosis or foam cells. Segmental sclerosis (S) was defined as any amount of the tuft involved with sclerosis, but not involving the whole tuft. Tubular atrophy (T) was defined by thick irregular tubular basement membranes with decreased diameter of tubules. It was scored according to the percentage of cortical area involvement, with 1%–5% rounded to 5% and other values rounded to the closest 10%. Interstitial fibrosis was defined as increased extracellular matrix separating tubules in the cortical area. It was scored as percentage involvement, with 1%–5% rounded to 5% and other values rounded to the closest 10% (11). Biopsies showing only sclerosed glomeruli were excluded from the study.

### Immunohistochemical analysis

Immunohistochemical staining for C4d was carried out on all cases on 3 µm sections of formaldehyde-fixed renal tissues. Patterns of positivity were recorded in the mesangium, glomerular capillary walls and peritubular capillaries. These patterns of positivity were scored according to the intensity. In glomerular deposition it was considered ‘global’ when >50% of the tuft was involved and ‘segmental’ when <50% of the tuft was stained for C4d. This was compared with the MEST score of Oxford classification to find correlation.

### Ethical issues

1) The research followed the tenets of the Declaration of Helsinki; 2) Informed consent was obtained; and 3) the research was approved by the Ethics Committee of Armed Forces Medical College, Pune, India (Ref: Ethics/STS/25/2014).

### Statistical analysis

The four parameters of Oxford classification were correlated with C4d deposition in various compartments of renal biopsies by Pearson correlation coefficient.

## Results

Of the 15 cases evaluated, 12 were males and 3 were females. The average age of the cases was 32 years.

**Figure 1:**
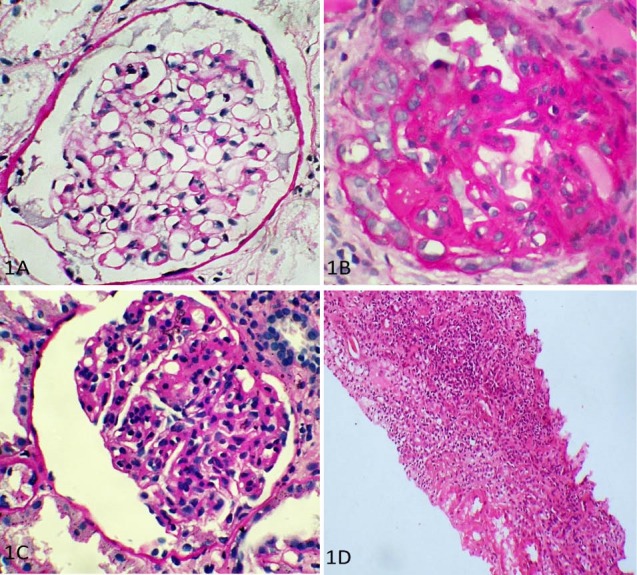
Spectrum of lesions according to Oxford classification for IgA nephropathy. (A) M0 E0 S0 (PAS stain, ×400); (B) M1 E1 S1 (PAS stain, ×400); (C) M0 E1 S0 (PAS stain, x400); (D) T2 lesion (H and E, ×100).

On histological examination, mesangial hypercellularity was found to be the commonest feature. Thirteen cases showed M1 lesions, 7 cases showed E1 lesions, 5 cases showed S1 lesions, and 2 cases each showed T1 and T2 lesions. On combining these scores, there were 2 cases which showed M0E0S0T0 morphology (Figure 1A), i.e., the glomeruli were essentially normal on light microscopy, but immunofluorescence confirmed the cases as IgAN. Four cases showed M1E0S0T0 morphology, indicating the presence of only mesangial proliferation, whereas two cases showed M1E1S1T1 scoring (Figure 1B). The rest of the cases showed various combinations of lesions (Figure 1C,  1D).

C4d deposition was found to be occurring mainly in mesangial location (12/15 cases, 80%). In the glomerular capillary walls, the deposition was present in segmental fashion in 6/15 cases (40%) and in global fashion in 4/15 cases (26.67%). Four (26.67%) cases showed deposition in the peritubular capillaries, 8 (53.33%) cases showed deposition in the arterioles and 3 (20%) cases showed deposition in the tubular epithelium. The intensity of staining ranged from 1 to 3 positive intensity.

**Figure 2:**
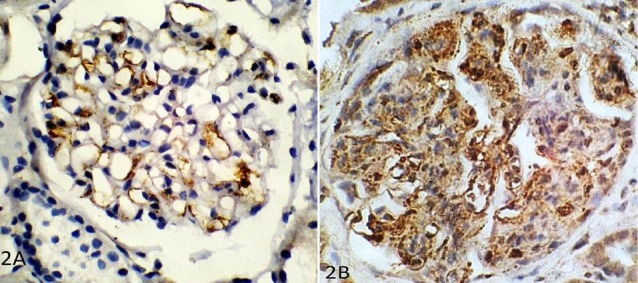
C4d immunohistochemistry showing deposition in glomerular capillaries in (A) segmental pattern, and (B) global pattern (×400).

The four parameters of Oxford classification were correlated with C4d deposition in various compartments of renal tissue by Pearson correlation coefficient. We found that segmental and global deposition of C4d in glomerular capillaries (Figures 2A, 2B) correlated best with endocapillary proliferation, i.e. E1 lesion of the MEST score (Table 1). Mesangial deposition did not significantly correlate with mesangial C4d deposition. Also, there was no significant association between C4d in the peritubular capillaries, tubular basement membranes and arterioles, and any of the four parameters of Oxford classification. Tubular atrophy and interstitial fibrosis, i.e. T lesions correlated significantly with the C4d deposition in the glomerular capillaries. However, T lesions correlated well with endocapillary proliferation as well and hence their association with C4d deposition can be explained.

**Table 1:** Pearson’s correlation coefficient analysis of various lesions in Oxford classification system and C4d deposition in various compartments of renal biopsies

**Statistical analysis**	**r**
M^a^ v/s C4d mesangial	- 0.100
E^b^ v/s C4d segmental capillaries	0.439
E v/s C4d global capillaries	0.255
S^c^ v/s C4d segmental capillaries	0.153
S v/s C4d global capillaries	0.243
T^d^ v/s C4d segmental capillaries	- 0.116
T v/s C4d global capillaries	0.514
T v/s C4d tubular epithelium	0.225
M+E v/s C4d mesangial + segmental + global capillaries	0.187
M+E v/s C4d PTC^e^	- 0.039
T v/s E	0.413

^a^Mesangial proliferation; ^b^Endocapillary hypercellularity; ^c^Segmental sclerosis; ^d^Tubular atrophy/interstitial fibrosis; ^e^Peritubular capillaries.

## Discussion

IgAN is an immune complex mediated glomerulonephritis. Since about 40% of cases are said to be progressing towards end stage renal disease, it is imperative to anticipate and identify such patients in order to treat them aggressively. Many studies have been carried out to establish clinical and histopathological features that can predict the outcome of this disease. Among the clinical parameters level of proteinuria, serum creatinine and presence of hypertension have been found to be associated with the outcome (12). However, the predictive outcome on the basis of histopathological features has remained controversial until recent past. The disease was morphologically classified by the Haas system till a few years ago. However, since the emergence of the Oxford classification system which generates the MEST score, all the research on this disease has been based on this system since it provides better reproducibility and comparability. Hence we chose to use the Oxford system of classification in this study.

C4d is a complement breakdown product and has been extensively studied in antibody mediated rejection. It has been included in the Banff criteria for diagnosing antibody mediated rejection in renal transplants (13). However, focus on its role in native proliferative glomerular diseases has come up only recently (6).

We found C4d deposition in the glomerular mesangium, glomerular capillary walls, peritubular capillaries and arterioles. However, deposition significantly correlating with parameters of the Oxford system was noted only in the glomerular capillary walls. In the study by Maeng et al, C4d deposition was described in 23 cases of IgAN (14). Their study showed glomerular deposition of C4d in thirteen cases (56.5%) and tubular epithelial deposition in 11 cases (47.8%). However, the authors did not further characterize the glomerular positivity as being mesangial or in the glomerular capillary walls. But they did describe the staining being more prominent in the higher classes of the World Health Organization (WHO) classification. They also reported an association of glomerular C4d staining with higher levels of albuminuria, an association we could not establish since we did not have the data in all the cases. However, we did have association of glomerular capillary wall staining with the histological parameter of endocapillary proliferation, which is known to clinically correlate with heavy albuminuria.

Espinosa et al studied mesangial C4d deposition in cases of IgAN and correlated it with the outcome (15). In their study, 40/87 cases (67.8%) were negative for C4d and out of the remaining 47 cases which were positive for C4d, 19 had pure mesangial deposition and 28 had mesangial glomerular capillary wall deposits. The renal biopsy showed that C4d-positive patients had significantly more glomerulosclerosis and a more severe interstitial fibrosis, and that a notably higher proportion evolved to ESRD in the follow-up. To analyse if C4d deposition was a consequence of chronic renal failure and glomerulosclerosis, this study was performed in 15 patients with nephroangiosclerosis. Renal biopsies of only two of them (13.3%) showed focal staining of C4d indicating that the deposition in the sclerosing glomeruli of IgAN was actually related to the disease process. However none of the above-mentioned studies have correlated C4d deposition with the Oxford classification which has been more widely accepted now.

Sahin et al had also studied glomerular staining of C4d in IgAN (16). They found that glomerulosclerosis and mesangial hypercellularity were present in most of the C4d-positive subjects and the difference was statistically significant. This is against our finding of correlation between C4d deposition and endocapillary proliferation. In addition, interstitial fibrosis/tubular atrophy were more severe in C4d-positive patients compared to C4d-negative patients, similar to our finding. They also found a correlation between urinary protein excretion and C4d deposition which we were unable to evaluate. In the logistic regression model, they found serum creatinine, glomerular filtration rate, proteinuria at the time of renal biopsy and C4d+ glomerular staining to be statistically significant contributors to the development of ESRD. In multivariate analysis only C4d-positivity and presence of hypertension were significantly associated with evolution to ESRD.

## Conclusion

We demonstrated deposition of C4d in the glomerular mesangium and along the glomerular capillary walls in segmental or global patterns. C4d deposition in the glomeruli correlated best with ‘endocapillary proliferation’ amongst all the four variables of the Oxford system that were analysed.

The deposition of C4d as demonstrated in this study supports the role of directed therapy against the complement system in cases of IgAN with severe glomerular manifestations in the form of endocapillary proliferation and C4d deposition in the glomeruli.

## Limitations of the study

There are some limitations to our study. The staining for C4d was performed by immunohistochemistry as against immunofluorescence which is considered by many to be more sensitive technique. However, considering that we obtained good staining in all of the cases, we considered our results satisfactory. We were unable to correlate the deposition of C4d with outcome in terms of progression to ESRD. The number of cases in our study was less and to further substantiate the correlation with each class of Oxford classification more number of cases in each class is required.

## Authors’ contribution

All authors contributed significantly to the work presented. AR and RT performed the study, and drafted the manuscript. SB and VSN along with AR and RT edited and reviewed the final manuscript. SM provided the clinical cases.

## Ethical considerations

Ethical issues (including plagiarism, misconduct, data fabrication, falsification, double publication or submission, redundancy) have been completely observed by the authors.

## Conflicts of interest

The authors declared no competing interests.

## Funding/Support

This research did not receive any specific funds from any group.
